# Protocol for a Type 3 hybrid effectiveness-implementation cluster randomized trial to evaluate multi-ethnic, multilevel strategies and community engagement to eliminate hypertension disparities in Los Angeles County

**DOI:** 10.1186/s13012-025-01452-5

**Published:** 2025-10-06

**Authors:** Sae Takada, Soma Wali, Nina Park, Atkia Sadia, Amelia R. Weldon, Li-Jung Liang, Stefanie D. Vassar, Savanna L. Carson, Alex R. Dopp, Ariella R. Korn, Alison B. Hamilton, Brian S. Mittman, Jocelyn Lo, Utpal Sandesara, Yu-Chuang Huang, Jessica Jara, Natalie Robles, Alejandra Casillas, Arleen F. Brown

**Affiliations:** 1 Division of General Internal Medicine and Health Services Research, Department of Medicine, David Geffen School of Medicine, University of California, 1100 Glendon Avenue, Suite 850, Los Angeles, CA 90024 USA; 2 Department of Medicine, Olive View-UCLA Medical Center, Sylmar, CA USA; 3 Department of Medicine, David Geffen School of Medicine, University of California, Los Angeles, CA USA; 4 Department of Population Health Management, Los Angeles County, Department of Health Services, Los Angeles, CA USA; 5 Department of Behavioral and Policy Sciences, RAND, Santa Monica, CA USA; 6 Department of Behavioral and Policy Sciences, RAND, Boston, MA USA; 7 Department of Psychiatry and Biobehavioral Sciences, David Geffen School of Medicine, University of California, Los Angeles, CA USA; 8 Center for the Study of Healthcare Innovation, Implementation, and Policy, VA Greater Los Angeles Healthcare System, Los Angeles, CA USA; 9 Health Services Research and Implementation Science, Department of Research and Evaluation, Kaiser Permanente Southern California, Pasadena, CA USA; 10 Olive View-UCLA Education and Research Institute, Sylmar, CA USA

**Keywords:** Hypertension disparities, Multiethnic interventions, Implementation facilitation, Safety net, Community engagement

## Abstract

**Background:**

In the U.S., racial and ethnic disparities in hypertension control contribute to disparities in cardiovascular mortality. Evidence-based practices (EBPs) for improving hypertension control have not been consistently applied across patient subgroups, especially in safety-net settings, contributing to observed disparities. The Los Angeles County Department of Health Services serves racially and ethnically diverse, low-income patients with hypertension and represents a valuable setting for research to reduce disparities. We designed a hybrid Type 3 effectiveness-implementation study using a three-arm, crossover randomized controlled trial to compare the effects of patient- and provider-focused strategies and usual implementation strategy on key implementation and clinical outcomes.

**Methods:**

We will enroll 27 primary care clinics. Patient-focused implementation strategies aim to increase patient access to culturally and linguistically tailored educational materials on hypertension and improve patient engagement in hypertension care. Provider-focused strategies include training in culturally tailored hypertension care and activities to strengthen clinic workflows for home blood pressure monitoring, medication titration, referral to nurse-directed blood pressure clinics, and social needs screening and referral. Implementation facilitators provide support for these EBPs. The primary implementation outcome is provider EBP adoption clustered at the clinic level, based on a scoring system using medical records, clinic observation, and webinar participation. The primary health-related outcome is the proportion of patients in a clinic with controlled hypertension by race and ethnicity. We will use the constrained generalized Poisson mixed-effects model to compare changes in event rate of provider EBP adoption between usual implementation strategy and either provider- or patient-focused strategies. We will use constrained logistic mixed-effects models to assess the effect on change in blood pressure control. We will record implementation progress using the Stages of Implementation Completion tool and identify costs and resource use using the Cost of Implementing New Strategies tool.

**Discussion:**

Our study contributes to the implementation science literature on cardiovascular health equity by examining alternative implementation strategies to increase use of culturally and linguistically tailored hypertension EBPs and social needs screening and intervention. Findings from our study will build evidence for implementation of hypertension EBPs in safety-net and other health systems serving racial and ethnic minority patients.

**Trial registration:**

Clinicaltrials.gov NCT06359691, registered April 10, 2024.

Contributions to the Literature

- Collaborating with patients, the healthcare system, and community members, we developed a three-arm, crossover randomized controlled study comparing patient-focused, provider-focused, and usual implementation strategies for the adoption of evidence-based practices (EBPs) in hypertension control within a safety net setting.
- Patient-focused strategies aim to improve access to culturally and linguistically tailored hypertension resources and engagement in care. Provider-focused strategies aim to teach culturally tailored care and strengthen team-based care and referrals. Implementation facilitators support clinic staff delivery of EBPs.
- Our study will provide evidence for implementing hypertension EBPs in safety-net and similar health systems serving racial and ethnic minority patients.

## Background

Hypertension is the leading cause of morbidity and mortality in the U.S. [1], leading to myocardial infarction, stroke, kidney disease, heart failure, and cognitive decline [2]. Nearly half of U.S. adults have hypertension, and only 1 in 4 adults with hypertension has adequate blood pressure control [3]. Racial and ethnic differences in hypertension prevalence, awareness, and control, along with their contributions to mortality disparities, are well-documented [4, 5]. Compared to White Americans, Black Americans have a higher prevalence of hypertension and lower rates of control, Latino Americans have similar prevalence but lower rates of treatment and control, and Asian Americans have similar prevalence and rates of treatment but lower rates of control [1], although limited disaggregated data are available for Asian American subgroups [6]. Underlying these racial and ethnic disparities are adverse social, economic, and political conditions that reduce educational and economic opportunities, access to healthy foods and safe spaces, and access to and quality of healthcare, while simultaneously producing chronic stress [7, 8]. The Los Angeles County Department of Health Services (LAC DHS) is a safety-net healthcare system that serves uninsured and publicly insured patients who often face such conditions [9, 10].

Evidence-based practices (EBPs) for improving hypertension diagnosis and management are widely available but have been inconsistently or suboptimally applied, contributing to health disparities. Such EBPs include utilizing hypertension registries, implementing treatment algorithms [11], promoting and supporting home blood pressure monitoring [12], offering culturally and linguistically tailored care and health education [13–16], and screening for and addressing social needs [17]. Implementation barriers for EBPs are especially pronounced for low-income and minority patients in safety-net settings [18]. Addressing racial and ethnic disparities by promoting implementation of hypertension EBPs in safety-net primary care settings requires multilevel, multi-component interventions to achieve an informed, activated patient and a prepared, proactive care team, as described in the Chronic Care Model [11, 19–21]. By providing tools to understand and reduce gaps in implementation of EBPs, implementation science – the study of methods for promoting systematic uptake of EBPs into routine care to improve quality of care [22] – offers promise for achieving health equity in cardiovascular disease [23].

Informed by the National Institute on Minority Health and Health Disparities (NIMHD) Research Framework [24], the Chronic Care Model [21], the Exploration, Preparation, Implementation, Sustainment (EPIS) framework [25, 26], and the Reach, Effectiveness, Adoption, Implementation, and Maintenance (RE-AIM) framework [27], this study compares patient- and provider-focused implementation strategies to facilitate adoption of an evidence-based multicomponent hypertension program in LAC DHS. Our program incorporates elements of the Kaiser Permanente Northern California hypertension program (henceforth “Kaiser Bundle”), a large-scale program to improve blood pressure control [11], which was successfully tailored for and implemented in San Francisco safety-net clinics [20]. Our program tailors and extends the Kaiser Bundle using community engagement techniques, which foreground integral, longitudinal partnership with groups of people most affected by research and interventions [28–30]. We also incorporate behavioral science approaches, which have been shown to increase uptake of EBPs by providers and healthy habits by patients [31–33] by leveraging existing social constructs and infrastructure, such as self-image, relationships, competition, and social norms [34–37].

We tailored multi-component hypertension EBPs and developed feasible implementation strategies for hypertension control within LAC DHS, explicitly addressing factors contributing to and maintaining racial and ethnic cardiovascular disease disparities. The primary implementation outcome is overall EBP adoption by providers clustered at the clinic level, to allow for implementation strategies and implementation facilitation to be directed at the clinic level. This article describes the resulting protocol for a three-arm, crossover randomized controlled trial to examine adoption, effectiveness, and sustainment of hypertension EBPs, comparing patient-focused, provider-focused, and usual implementation strategies in LAC DHS primary care clinics. We hypothesize that, compared to usual implementation strategies, provider- or patient-focused implementation strategies will produce higher rates of EBP adoption clustered at the clinic level (primary implementation outcome) and improve overall blood pressure control clustered at the clinic level (clinical effectiveness outcomes), with additional benefits in secondary outcomes, including reduced hypertension disparities and increased health system engagement with community organizations and resources. We further hypothesize that these changes will be sustained for at least one year after study’s active implementation phase concludes.

## Methods

### Study Setting

The study will be conducted in 27 LAC DHS clinics that provide adult primary care. LAC DHS is the nation’s second-largest municipal health system, providing care for 750,000 unique patients annually through an integrated system of four hospitals and 24 health centers [38]. LAC DHS clinics serve racially and ethnically diverse, socioeconomically underserved patients, many with hypertension. In 2024, among 455,672 adult primary care patients in LAC DHS, 82,525 (18%) had a hypertension diagnosis, including 54,481 Latino Americans, 9,474 Black Americans, 2,927 Filipino Americans, 673 Chinese Americans, and 601 Korean Americans. Among patients with hypertension, 33% have poor control (systolic blood pressure > 140 or diastolic blood pressure > 90 [39]), ranging from 23% of Chinese Americans to 42% of Black Americans (Table 1).

**Table 1:** Demographic characteristics of primary care patients with hypertension at Los Angeles County Department of Health Services clinics enrolled in the study

		**Total** **(n = 82,525)**	**Hispanic/Latino American** **(n = 54,481)**	**Black American** **(n = 9,474)**	**White American** **(n = 3,094)**	**Filipino American** **(n = 2,927)**	**Chinese American** **(n = 673)**	**Korean American** **(n = 601)**	**South Asian American** **(n = 469)**	**Other** **(n = 10,808)**
**Mean Age (SD)**		59 (12)	59 (12)	58 (12)	59 (11)	63 (12)	63 (10)	60 (10)	62 (11)	57 (12)
**Gender**	**Female**	44736 (54.2%)	30769 (56.5%)	4979 (52.6%)	1312 (42.4%)	1677 (57.3%)	411 (61.1%)	345 (57.4%)	237 (50.5%)	5006 (46.3%)
	**Male**	37768 (45.8%)	23701 (43.5%)	4491 (47.4%)	1781 (57.6%)	1250 (42.7%)	262 (38.9%)	255 (42.4%)	232 (49.5%)	5796 (53.6%)
Hypertension Control*	**Controlled**	54412 (65.9%)	36694 (67.4%)	5404 (57.0%)	2087 (67.5%)	2096 (71.6%)	503 (74.7%)	428 (71.2%)	341 (72.7%)	6859 (63.5%)
	**Inadequately controlled**	27095 (32.8%)	17377 (31.9%)	3953 (41.7%)	952 (30.8%)	792 (27.1%)	157 (23.3%)	161 (26.8%)	119 (25.4%)	3584 (33.2%)

*Hypertension Control defined as systolic blood pressure <140 and diastolic blood pressure < 90

### Conceptual frameworks

The NIMHD Research Framework and the Chronic Care Model guide our identification of the root causes of observed disparities and our design of clinical intervention bundles and implementation strategy bundles. The NIMHD Research Framework conceptualizes the complex and multifaceted nature of minority health and health disparities by identifying various domains of influence (biological, behavioral, physical/built environment, sociocultural environment, healthcare system) and levels of influence (individual, interpersonal, community, societal) [24]. The Chronic Care Model illustrates ways to improve management of chronic conditions by modifying delivery system, decision support, clinical information systems, healthcare organization, self-management support, and community resources to achieve an informed, activated patient and a prepared, proactive care team. Based on these frameworks, we selected implementation strategies that target multiple domains of influence at various levels, and engage the healthcare system, care team, patients, and local community to address disparities. For example, the culturally and linguistically tailored patient education materials support patient self-management of hypertension while incorporating communication with and navigation of family, social structures, care teams, and healthcare system to teach about hypertension, healthy lifestyle, home monitoring, and medication adherence.

We use the EPIS framework to further guide the implementation process [26, 40]. EPIS describes four phases (Exploration, Preparation, Implementation, and Sustainment) and identifies key determinants influencing implementation, organized within four domains. During the three-year planning stage, we examined the domains – Outer Context (communities where patients live), Inner Context (healthcare systems and clinics), Innovation Factors (hypertension EBPs), and Bridging Factors (system links to external partners) – for the Exploration phase (identifying needs, selecting hypertension EBPs) and Preparation phase (identifying implementation determinants, planning for implementation of hypertension EBPs). The Type 3 hybrid trial focuses on Implementation (initiating and monitoring implementation of hypertension EBPs) and Sustainment (embedding hypertension EBPs with ongoing support and monitoring).

### Study development

This study is funded by the U.S. National Heart, Lung and Blood Institute, National Institutes of Health, as part of the Disparities Elimination through Coordinated Interventions to Prevent and Control Heart and Lung Disease Risk (DECIPHeR) Alliance [26]. In partnership with LAC DHS and community partners, our multidisciplinary team developed the study protocol during a three-year planning stage designed to tailor multilevel EBPs to clinical and community contexts and design implementation bundles. During this phase, we undertook a systematic literature review on hypertension interventions in multi-ethnic communities [41]. To assess EBPs identified in the systematic review and understand barriers and facilitators to their implementation, we formed a steering committee comprising representatives from community, health system, and academic sectors and organized working groups with LAC DHS leadership. To understand community perspectives on hypertension management and obtain recommendations for culturally tailoring EBPs, we recruited five ethnicity-specific Community Action Boards (CABs) representing Latino, Black, Filipino, Chinese, and Korean American communities [42]. To inform implementation strategy design and tailoring, we conducted and analyzed interviews and focus groups with diverse health system personnel, community-based organizations, and safety-net patients. We iteratively refined study aims, along with EBP and implementation bundle design, and strengthened research design and analytic plans through Technical Assistance meetings with National Institutes of Health statisticians [43] and LAC DHS leadership. We modified the study design from a stepped-wedge to a three-arm, crossover design to reflect LAC DHS commitment to equitable access to resources for all patients. As part of the national DECIPHeR network, we actively participated in all subcommittees (including Implementation, Intervention, Community Engagement, Design and Analysis) and multi-site manuscript preparation [26]; developed an integrated implementation determinant framework for shared learning of community-engaged health equity implementation research [44, 45]; contributed to a paper on community engagement strategies in implementation science [46]; and developed a meta-framework for implementation science that centers equity and community engagement to help inform equitable implementation measures and strategies [47].

### Overview of trial design

This study uses a three-arm, cluster randomized crossover design to conduct a Type 3 hybrid effectiveness-implementation trial comparing the impact of different sequences of patient-focused and provider-focused strategies (all supported by implementation facilitation) on EBP adoption clustered at the clinic level. A hybrid Type 3 study aims to measure the effects of an implementation strategy bundle while simultaneously assessing the implemented intervention’s effectiveness in improving clinical outcomes [48].

**Fig. 1:**
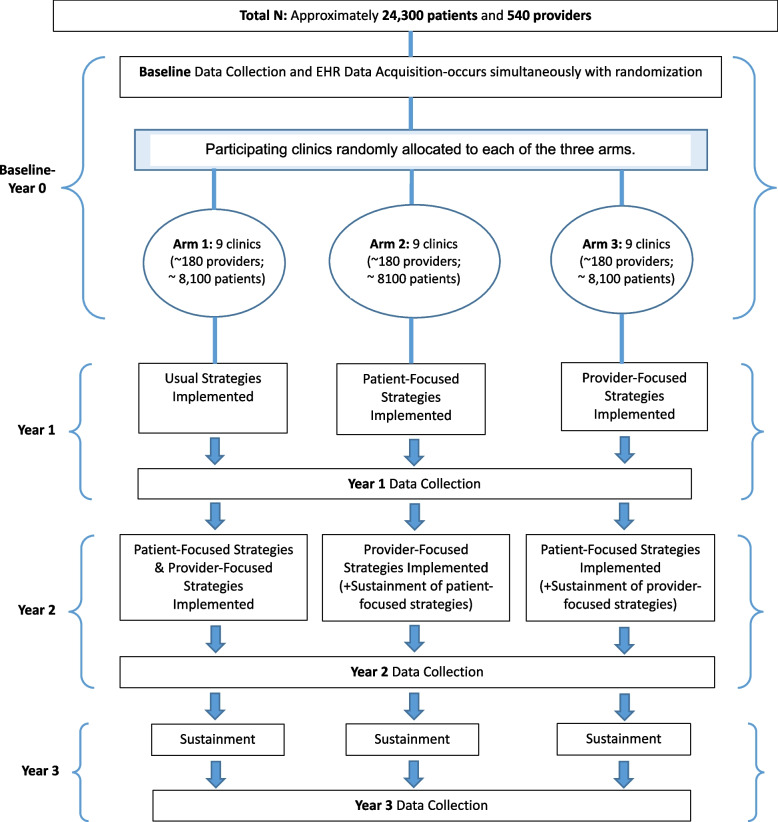
Three-arm, cluster randomized crossover design

The three arms receive (see Fig. 1):

- Arm 1: Usual implementation strategies in Year 1 followed by both patient-focused and provider-focused strategies in Year 2
- Arm 2: Patient-focused strategies in Year 1 followed by provider-focused strategies in Year 2
- Arm 3: Provider-focused strategies in Year 1 followed by patient-focused strategies in Year 2

Table 2 lists our study aims and hypotheses. The trial was registered in ClinicalTrials.gov (NCT06359691) and was approved by the Olive View-UCLA Education and Research Institute Institutional Review Board (#2,097,481), with reliance agreements with UCLA (#24–5057), Los Angeles County Department of Public Health (#10,136), RAND (#2024-N0071), and University of Southern California (#UP-24–0093).

**Table 2:** Primary and Secondary Study Aims

Aim Type	Aim	Hypothesis
Primary Implementation Aim	To test the effectiveness of the implementation strategies (usual implementation, patient-focused, and provider-focused) on change in adoption of EBPs at end of Year 1	Compared to usual implementation strategy (Arm 1), provider- or patient-focused implementation strategies (Arms 2 or 3) will exhibit greater increase in overall provider adoption of EBPs
Secondary Implementation Aim 1	To examine the impact of the sequence of the implementation strategies (usual implementation followed by both provider- and patient-focused; patient-focused followed by provider-focused; provider-focused followed by patient-focused) on change in EBP adoption at end of Year 2	Provider-focused followed by patient-focused implementation strategies (Arm 3) is expected to show greater increase in provider adoption of EBPs compared to usual implementation followed by both (Arm 1)
Secondary Implementation Aim 2	To examine the impact of the sequence of the implementation strategies on secondary implementation outcomes of EBP acceptability, appropriateness, and feasibility	Provider-focused followed by patient-focused implementation strategies (Arm 3) compared to usual implementation followed by both (Arm 1) is expected to show greater increase in secondary implementation outcomes
Health-Related Aim 1	To test the effect of the implementation strategies (usual, patient-focused, and provider-focused) on change in HTN control at end of Year 1	Provider- or patient-focused strategies (Arms 2 or 3) are expected to show greater increase in patient’s HTN control, compared to usual implementation (Arm 1)
Health-Related Aim 2	To examine the impact of the sequence of the implementation strategies on secondary health-related outcomes of HTN control, medication adherence, and disparities in HTN control at end of Year 2	Provider-focused followed by patient-focused implementation strategies (Arm 3) compared to usual implementation followed by both (Arm 1) is expected to show greater improvement in health-related outcomes

### Implementation facilitation

We will use implementation facilitation as an overarching strategy to support EBP adoption. Facilitation is a process of interactive problem-solving and support by skilled individuals to enable others to adopt EBPs and has been found to enhance EBP adoption in many settings, including primary care [49–51]. Facilitation support at each clinic will be led by study staff serving as external facilitators and LAC DHS nursing staff serving as internal facilitators. The external and internal facilitators will work in pairs to build relationships with assigned clinics, gather ongoing input to inform implementation, and guide delivery of provider- or patient-focused strategies. Facilitators will work closely with each clinic’s leadership and staff, including “Clinic Champions” designated by leadership to help guide local efforts to implement and sustain hypertension EBPs. Facilitators will receive standardized training in implementation facilitation [50] alongside project-specific training on patient- or provider-focused strategies. Once trained, facilitators will deliver the same type of strategy (patient- or provider-focused) for the entire trial, changing the clinics they work with based on the strategies to which the clinics are assigned. Facilitators will receive ongoing oversight and support from a Facilitator Support Team of implementation experts and project managers. The following describes key facilitation-related roles.

#### External facilitator

External facilitators will lead planning and execution of clinic site visits, meetings, and other activities designed to support implementation (e.g., HeartBEAT learning collaborative) [42]. To understand how EBPs are being implemented and how to better support clinics and patients, external facilitators will track implementation progress using the Stages of Implementation Completion (SIC) tool [52], described below. Quarterly, external facilitators will collaborate with Clinic Champions to complete a fidelity checklist that monitors EBP intervention fidelity and guides improvements over time. For clinics where implementation progress for EBP uptake is low, external facilitators will identify and train site-specific champions to serve as additional facilitators who assist with efforts; previous research has demonstrated this approach’s efficiency in implementation facilitation [53]. For external facilitators, weekly Facilitator Support Team meetings alternate between in-depth progress updates focused on key SIC milestones and broader reflections on high-level successes, challenges, and growth areas [54].

#### Internal facilitator

As health system employees, internal facilitators will leverage their clinical expertise to support clinics’ use of patient- or provider-focused strategies. Internal facilitators will help identify and troubleshoot barriers within DHS referral pathways and support additional facilitation activities in collaboration with the external facilitators (e.g., HeartBEAT meetings and plan-do-study-act [PDSA] cycles in the provider-focused condition). Internal facilitators work more limited hours than external facilitators and cannot routinely attend Facilitator Support Team meetings, but regular contact with their external facilitator partners and support team members will ensure they receive adequate role-specific support [55].

### Study participants

#### Clinics

All LAC DHS adult primary care clinics will participate in the trial over the full three-year timeline. We anticipate 27 participating LAC DHS clinics, and plan to recruit an average of 20 providers and 900 patients with hypertension per clinic. The 27 clinics will be grouped into nine sets of three clinics each based on clinic size (large, medium, small) and geographic location as defined by Los Angeles County Service Planning Area [56] (Antelope Valley and San Fernando Valley; San Gabriel Valley and East; Metro, South, and South Bay). Within each set, a masked study statistician will randomize clinics to a study arm using SAS Software Proc Plan [57].

#### Providers

We will survey health care providers at baseline and after Years 1, 2, and 3. Providers include those providing care to patients with hypertension: physicians, nurses, advanced practice providers, social workers, health educators, behavioral health workers, community health workers (CHWs), and pharmacists. (CHWs are community members employed by the health system and trained as patient and community advocates, disease management coaches, and health system navigators for medically and socially complex patients [58, 59]). At each clinic, medical directors (or their designees) will identify a list of potential provider participants. We will sample the list to get representation from different provider types within the clinic. We anticipate surveying approximately 540 providers across the 27 clinics. Medical directors (or their designees) will announce the study at staff meetings and via emails. The research team will provide informational flyers about the study. We will obtain informed consent for the survey. Providers will receive a $75 gift card for completion of each survey.

#### Patients

We will extract electronic health record (EHR) data for eligible patients with a baseline diagnosis of hypertension, empanelment (assignment) to primary care providers in participating clinics, and at least one visit and one recorded blood pressure measurement within the past 12 months, consistent with National Committee for Quality Assurance (NCQA) Healthcare Effectiveness Data and Information Set (HEDIS) specifications [39]. We will extract EHR data at baseline and after Years 1, 2, and 3. We will not contact, consent, or incentivize patients.

### Implementation strategies

We will use patient- and provider-focused strategies to facilitate use of tailored EBPs to improve the management of hypertension in primary care. The tailored EBPs are based on the Kaiser Bundle [11] and additional practices relevant to our study context. Table 3 describes the EBPs, their adaptations for the study, and the patient- and provider-focused strategies aimed at encouraging EBP uptake.

**Table 3:** Provider- and Patient-focused Implementation Strategies for Evidence-based practices

Evidence-based practice	Adaptations	Usual Strategies	Provider-Focused Strategies	Patient-Focused Strategies
1. **Hypertension Registry (Kaiser Bundle)** Clinics use the HTN registry to identify patients with inadequately controlled BP or no PCP appointment in the last 3 months, send reminders to the patient to schedule a PCP appointment, and notify care teams	Not Applicable	A HTN registry is not used routinely for patient or provider communication	Study team provides Clinic Champions with a list of patients with uncontrolled HTN who have had no PCP appointment in the last 3 months and no PCP appointment scheduled in the next 3 months	Study team identifies patients in the HTN registry with uncontrolled BP, no PCP appointment in the last 3 months, and no PCP appointment scheduled in the next 3 months, and sends reminders to the patient to schedule a PCP appointment Patients on the HTN registry may receive a link to culturally- and linguistically-tailored HTN educational materials via text message or the patient portal
2. **Home Blood Pressure Monitoring** Clinics utilize a standardized workflow to provide home BP monitors to all patients with HTN	This EBP was included to reflect updated guidelines on home BP monitoring, staff input, and changes in health plan policies	Not all providers are familiar with the workflow for ordering home BP monitors through insurance for eligible patients. Logistical barriers, such as backordered monitors, have been reported. For patients without insurance, free home BP monitors are provided through a patchwork of funding sources	Provider webinars (PCP, CHW, Nurse) describe standardized workflows for providing BP monitors to eligible patients through prescription or referral to the health plan and for reviewing and documenting home BP readings through the patient portal	Patient educational materials encourage patients to obtain a home BP monitor and how to ask a provider about ordering one. Once they have a monitor, patient is taught how to measure and log their BP, either in the patient portal or on a paper log they bring to clinic
3. **Standardized Blood Pressure Measurement (Kaiser Bundle)** Clinics use a standardized protocol for taking BP and have trained clinicians on this protocol	This EBP was adapted to incorporate standardized measurements at home, reflecting updated home BP monitoring guidelines	No specific implementation process exists for standardizing BP measurement across clinics and providers	Provider webinars describe how to use standardized BP measurement. Illustrated posters outlining standardized BP measurement practices are posted in clinic rooms where vitals are taken	Patients who receive home BP monitors receive training on standardized BP measurement practices Patient educational materials describe how to measure BP at home
4. **Treatment Intensification Protocol with Fixed Dose Combination Medications (Kaiser Bundle)** Patients with high BP are treated using fixed-dose combination pills, which can improve medication use, adherence, and BP control	This was adapted to reflect LAC DHS updated formulary. Based on clinician input, we developed provider webinar on fixed-dose combination medications	LAC DHS developed an HTN Expected Practices guide based on national guidelines, but uptake varies by clinic. Providers described challenges in interpreting multiple national guidelines. Intensification protocols and fixed-dose combination medications are not specifically emphasized in the Expected Practices	Provider webinars increase awareness of the efficacy and availability of combination medications in the formulary and LAC DHS HTN Expected Practices	Patient education materials describe HTN medications, including fixed-dose combination pills. Materials frame normalization and efficacy of combination medications and provide accurate information regarding common medication myths
5. **Nurse-directed Blood Pressure Clinics (Kaiser Bundle)** Providers can refer patients to a nurse-directed BP clinic within their clinic or at another clinic. Providers know how to refer patients, and there is a workflow for scheduling patients in those clinics	This EBP was adapted from pharmacist-directed to nurse-directed clinics to address a shortage of pharmacists and to incorporate existing nurse-directed clinics. We also added a CHW and Health Educator role to promote adherence	Some clinics have implemented nurse-directed BP clinics through pilot grants. There is variation in use among individual providers. The program is being scaled to other clinics	Provider webinars describe how to refer eligible patients to nurse-directed BP clinics	Patient educational materials inform patients about the nurse-directed BP clinic for supportive counseling on medications, dose adjustments, or concerns
6. **Culturally and Linguistically Tailored Education Materials** Patients with HTN receive culturally and linguistically tailored education materials at the end of the visit. These materials are available electronically for patients and providers	This EBP was adapted to include staff training on culturally responsive care and culturally and linguistically congruent outreach to patients	Culturally- and linguistically- tailored resources exist but have not been systematically circulated and organized. Care teams lack a straightforward way to access all relevant resources	Provider webinars describe the availability of culturally and linguistically tailored patient educational materials available on LAC DHS website, internal communication platforms, and printed in clinic. Provider webinars teach culturally responsive HTN care	Culturally and linguistically tailored educational materials may be sent to patients via text, patient portal, or hard copy in clinic/mail
7. **Screening for Social Needs and Linkage to Community Resources** Clinics systematically screen for social needs and refer patients to the Behavioral Health Integration Team for linkage to resources within their community	This EBP was adapted to include culturally relevant local resources for a variety of racial and ethnic groups	Social needs screening is embedded into standard primary care visit protocols, but there is varied awareness and understanding of the screening and referral processes. Once a patient screens positive and is referred to the Behavioral Health Integration Team, providers receive limited communication	Provider webinars educate providers on how to screen and refer patients to community resources	Patient educational materials discuss the value of community resources to meet social needs, including how to make self-referrals to some services
8. **Community Health Workers for Patients with HTN *and* Complex Medical and Social Needs** Clinics have a procedure in place to identify patients and refer them to the Behavioral Health Integration Team for linkage to CHWs	Not Applicable	CHWs are available system-wide to help address social needs and support engagement in medical treatment, but referrals to CHWs is inconsistent between providers and clinics. Variable integration of CHWs across different clinics results in their role being underutilized	Provider webinars educate providers on how to refer socially and medically complex patients to CHWs. Webinars for CHWs describe evidence-based and culturally relevant HTN care	Patient educational materials discuss the value of community resources to meet social needs
9. **Behavioral science to encourage behaviors related to HTN care** Behavioral science considers the environmental, social, and personal factors that impact how a person chooses to act	Not Applicable	Not Applicable	Clinics display posters to promote available resources and best practices for HTN care in provider charting and break rooms	Clinics display posters to promote best practices for HTN self-management in patient waiting areas and examination rooms. Messages sent to patients with uncontrolled HTN enhance patient engagement using behavioral science

*EBP* Evidence-based practice, *PCP* Primary Care Provider, *BP* Blood pressure, *HTN* Hypertension, *CHW* Community health worker, *LAC DHS* Los Angeles County Department of Health Services

The Kaiser Bundle includes a hypertension registry, standardization of blood pressure measurements, an evidence-based treatment protocol using fixed-dose combination medications, and medical assistant-led blood pressure clinics for medication adjustments. The Kaiser Bundle has been adapted and implemented in San Francisco safety-net clinics serving a racially and ethnically diverse patient population, where it improved blood pressure control across all racial and ethnic groups [20].

During the planning stage, we made several adaptations to the Kaiser Bundle. We incorporated home blood pressure monitoring (EBP #2); formulary additions to increase availability of combination medications (EBP #4); nurse-directed blood pressure clinics (EBP #5), culturally and linguistically tailored education materials (EBP #6); screening for unmet social needs like food insecurity, housing instability, and justice-involvement alongside linkage to corresponding health system and community resources (EBP #7); and CHW referral for medically and socially complex patients with high utilization or poor clinical outcomes (EBP #8). Our modifications were based on system-level barriers and potential facilitators identified by patients, providers, staff, and community members (e.g., culturally and linguistically tailored education, addressing social needs through community resources and CHWs); emerging evidence (e.g., home blood pressure monitoring recommended by new guidelines [12]); and practical considerations (e.g., availability of nurses for blood pressure clinics [60, 61], which LAC DHS was already introducing when we developed our project).

### Patient-focused strategies

Patient-focused strategies are designed to enhance patients’ access to culturally and linguistically tailored hypertension care resources and promote engagement in care.

#### Patient education materials

We developed patient educational materials based on American College of Cardiology/American Heart Association (ACC/AHA) guidelines, publicly available hypertension education resources, behavioral science, LAC DHS clinical guidelines, behavioral and cultural tailoring frameworks, and a rigorous iterative review process involving multiple partners. We incorporated insights from patient focus groups, CAB meetings, and healthcare personnel interviews conducted in the planning stage, which highlighted the value of addressing hypertension basics, common myths and misconceptions, medication adherence barriers, home blood pressure measurement techniques, and cultural and linguistic tailoring. We culturally tailored materials in consultation with patient focus groups, health system personnel, and ethnicity-specific CABs [42], and linguistically tailored materials using a translation vendor, followed by review by local native speakers. Four culturally concordant, multilingual graphic designers designed the finished products.

To date, we have developed 43 culturally and linguistically tailored patient education materials for five communities in English, Chinese (Simplified and Traditional), Korean, Spanish, and Tagalog. Education materials are designed to help patients understand hypertension, encourage home blood pressure measurement, promote blood pressure-lowering lifestyle habits, address medication-related concerns, increase awareness of clinic resources, and highlight health system resources for social needs (Table 3). To satisfy diverse learning needs, written materials were designed in several formats: comprehensive interactive handout packets, high-yield infographics, and informational posters for clinic waiting areas and exam rooms. Materials will be available on LAC DHS’s website. In the patient-focused strategy arm, patients will receive materials via patient portal, text message, or mail, per individual preference.

#### Videos

We also produced 2- or 3-min videos of patients describing lived experiences with hypertension. These can be played in clinic waiting rooms and placed on LAC DHS’s website.

#### Patient outreach

Patients with uncontrolled hypertension empaneled to clinics in the patient-focused strategy arm will receive messages through the patient portal or via text message. All messages contain a link to the culturally and linguistically tailored hypertension education materials described above. In addition, patients who have no primary care or nurse-directed hypertension clinic appointment scheduled within the next three months, or missed a primary care or nurse-directed hypertension clinic appointment within the last three months, will receive the following message: “Your care team needs to check your blood pressure and has saved an appointment for you. This visit is important to keep you healthy. Call *[clinic phone number]*to schedule your appointment within the next 3 months.” By stating, “Your care team has saved an appointment for you,” this message aims to increase clinic visits based on behavioral science insights that telling patients something is reserved for them enhances perceptions of selectivity and influences choice [62, 63]. We will send a follow-up message one week later, with up to three total messages per patient. For patients who do not complete a blood pressure management appointment after three messages, internal facilitators will conduct phone outreach to support scheduling.

### Provider-focused strategies

Care teams in clinics assigned to the provider-focused strategies will receive training to increase awareness about EBPs and provide guidance in their use (Table 3).

#### Provider webinars

We developed role-specific provider webinars for physicians, advanced practice providers, nurses, medical assistants, behavioral health providers, and CHWs. To design webinars, we collaborated with health system leaders with expertise in hypertension and health professions education. Webinars incorporated content from the ACC/AHA hypertension guidelines [12, 64] and LAC DHS expected practices. We tailored webinars using input from patients, ethnicity-specific CABs, and health system personnel, which underscored how social determinants, health literacy, and medical trust shape hypertension management. Informants also emphasized the value of culturally responsive care and recommended robust linkage to resources for unmet social needs [42]. Providers recommended clarifying nuances relating to evidence-based lifestyle recommendations, overlapping national guidelines, and therapeutic inertia. Patients recommended strategies to tackle adherence barriers, such as motivational interviewing, consideration of family context or social norms, and explanations of medications’ origins for those preferring holistic therapy.

To date, we have developed six webinars, including four 60-min sessions for physicians and advanced practice providers, one 60-min session for nurses, and one 60-min session for behavioral health staff and CHWs. These webinars are designed to educate providers on hypertension disparities within the health system, standardized guidelines for office-based blood pressure measurements, evidence-based lifestyle modifications, and fixed-dose combination medications on the LAC DHS formulary, along with delivery of evidence-based, culturally tailored hypertension care in their respective roles. Webinars also highlight clinic workflows, such as referrals for insurance-covered home blood pressure monitors, referrals to nurse-directed blood pressure clinics, and social needs screenings with linkages to CHWs, health educators, and community resources.

A peer champion will lead provider webinars over video conference within the first few months of provider-focused strategy initiation. Webinar completion is encouraged thorough endorsements by system leadership, email reminders, and continuing education credits where applicable. Following behavioral science insights on the motivational power of peer comparisons, we will recognize clinics for rates of webinar participation midway through the intervention year. Module recordings and slides will remain available through LAC DHS’s online training platform and commonly used communication platform.

#### Learning collaborative

All clinics receiving provider-focused strategies will be invited to join a learning collaborative [65], called “HeartBEAT (Blood pressure Equity Action Team),” organized by external facilitators. Learning collaboratives gather representatives from multiple implementation sites for shared learning and problem-solving. In HeartBEAT, quarterly meetings of clinic champions and leadership will focus on how clinics are working toward hypertension equity; between meetings, clinics will use quality improvement strategies like PDSA cycles to enact desired practice and policy changes. Furthermore, nurse facilitators will offer provider-focused technical assistance through monthly office hours.

#### Blood pressure care clinic champion

The Blood Pressure Care Clinic Champion is a staff member who becomes a clinic expert in best practices for implementing evidence-based, culturally tailored, equitable hypertension care within LAC DHS and serves as a liaison between the clinic and the study to support clinic change. The Champion will give clinic leadership regular project updates, meet monthly with the clinic’s assigned external or nurse facilitator, distribute and monitor use of culturally tailored materials, contribute information to support quarterly completion of the intervention fidelity checklist, participate in HeartBEAT meetings to share learnings on EBP delivery, and disseminate lessons among the clinic’s staff and leadership.

#### Resource guide and provider-focused clinic poster

We developed written materials to reinforce EBP adoption. Posters placed in clinics in the provider-focused strategy arm summarize tools for EBPs. A Resource Guide highlights local resources and summarizes step-by-step workflows for LAC DHS. As with posters developed for the patient-focused strategies arm, the provider-focused posters incorporate behavioral science by promoting social norms of hypertension care.

### Outcomes

We identified key implementation outcomes using the RE-AIM [66] framework (Table 4). The primary implementation outcome is overall provider EBP adoption clustered at the clinic level, determined using a scoring system derived from EHR data, LAC DHS administrative data, LAC DHS Patient Outreach Center data, the patient portal, and registration logs for hypertension training sessions. The main health-related outcome will be proportion of patients within a clinic with controlled hypertension by race and ethnicity. The definition of adequate blood pressure control is blood pressure < 130/80 [69]. We will also use an alternative, more stringent threshold, such as blood pressure < 120/80, since studies suggest that patients with established atherosclerotic cardiovascular disease assigned to lower targets have lower rates of cardiovascular events or death compared to those assigned to standard targets [67, 70, 71]. The data source will be LAC DHS’s EHR. Finally, we will track community engagement activities and new collaborations through discussions with and surveys of LAC DHS leadership and managers, Steering Committee members, and CAB members.

**Table 4:** Implementation and Effectiveness Outcome Measures, Data Sources, and Data Collection Time Points

Domain	Measure	Target	Data Source	Timing During Implementation			
					**Implementation**		**Sustainment**
				**Year 0**	**Year 1**	**Year 2**	**Year 3**
**Reach**							
Patient population coverage	% of eligible clients receiving EBP^a^		EHR	**✓**	**✓**	**✓**	**✓**
Disparities in coverage	Rates of EBP receipt by patient race and ethnicity		EHR	**✓**	**✓**	**✓**	**✓**
**Effectiveness**							
BP control^b^	AHA/ACC Guidelines		EHR	**✓**	**✓**	**✓**	**✓**
Secondary outcomes^b^	AHA/ACC Guidelines		EHR	**✓**	**✓**	**✓**	**✓**
**Adoption**							
Provider adoption^a^	See scoring system (Table 6)	Provider	EHR and Online Provider Survey	**✓**	**✓**	**✓**	**✓**
**Implementation**							
Acceptability, appropriateness, and feasibility^b^	Adapted Acceptability of Intervention Measure (AIM); Intervention Appropriateness Measure (IAM); Feasibility of Intervention Measure (FIM) [43]	Provider	Online Provider Survey, Periodic Reflections	**✓**	**✓**	**✓**	**✓**
Delivery and quality of implementation strategies^b^	Stages of Implementation Completion (SIC) [67]	Provider	SIC dashboard, Periodic Reflections		**✓**	**✓**	**✓**
Implementation costs (e.g., provider time, incentives, training, EHR updates)^b^	Costs of Implementing New Strategies (COINS) [68]; other Cost Instruments	Provider	SIC AND COINS dashboard		**✓**	**✓**	
**Maintenance/Sustainment**							
Clinician intent to maintain intervention component^b^		Provider	Online Provider Survey			**✓**	
Sustainment of reach, adoption, effectiveness, implementation outcomes^b^		Provider	Online Provider Survey				**✓**

*EBP* Evidence-based practice, *BP* Blood pressure, *EHR* Electronic health records, *AHA*, American Heart Association, *ACC* American College of Cardiology

^a^ Primary Outcome

^b^ Secondary Effectiveness Outcomes include health-related outcomes, disparities, and sustainment

### Data collection

#### Masking

We developed a detailed masking protocol that describes data access for different research team members (Principal Investigators, Co-Investigators, Project Coordinator and Director, Facilitators, Statisticians, Data Managers, and Data Analysts). Data includes clinic and provider study arm assignment, clinic-level stages of implementation, EHR data, provider surveys, and clinic-, provider-, and patient-level administrative data (e.g., webinar participation). For example, an unmasked data analyst who knows study arm assignment of clinics will obtain, clean, and harmonize EHR data, while a separate masked statistician/data analyst will conduct analyses for primary and secondary outcomes.

#### Provider survey

The annual provider survey begins with demographic characteristics, training, years worked in the clinic, and overall impression of the clinic’s approach to hypertension management. Subsequent sections ask about the eight EBPs. Each section describes an EBP before asking the provider whether they followed the EBP in the past 12 months, how successful EBP implementation was, and how often they expect to follow the EBP in the next 12 months; each section also solicits ratings for acceptability, appropriateness, and feasibility of the EBP, using questions adapted from the Acceptability of Intervention Measure, Intervention Appropriateness Measure, and Feasibility of Intervention Measure, respectively [68].

#### Clinic EHR

We will extract annual, de-identified blood pressure data from the EHR, using NCQA HEDIS specifications, for eligible adult patients (> 18 years) with hypertension seen in participating clinics [39]. We will collect date, location, time, and value of measurement. In cases with multiple measurements on one day, we will record them all and average the values [72].

#### Implementation progress

External facilitators will monitor and record progress toward implementing EBPs using the SIC [52], a web-based tool for monitoring implementation delivery and quality that can be customized to track relevant activities. The SIC will track dates and completion of activities across Implementation (staff hired and trained, fidelity monitoring, consultation, ongoing services) and Sustainment phases. The SIC allows facilitators to track progress and actively intervene when concerns arise, as implementation is more likely to be successful when activities are completed thoroughly and quickly. The SIC will provide metrics of implementation progress for each clinic, such as number of implementation activities completed, which will help contextualize primary and secondary implementation outcomes.

#### Intervention-related costs

We will use the Cost of Implementing New Strategies (COINS) [73], a tool embedded within the SIC, to map costs and resource use (e.g., personnel time) onto implementation activities. When analyzing COINS data, we will work with LAC DHS leadership to establish appropriate personnel and resource costs. For example, facilitators will track time spent on various activities, and LAC DHS will provide hourly rates for that time by role. We will summarize implementation costs by trial arm.

#### Partnership sustainment and community engagement

For our quarterly Steering Committee Meetings, weekly LAC DHS Health System Partnership meetings, and quarterly CAB meetings, we will document discussions, including feedback on implementation strategies, progress, challenges and successes, reflections on partnerships, sharing of study-generated patient and provider materials, and interpretation and dissemination of results.

#### Data management

Clinic-level data will be encrypted and stored on a secure UCLA server, while patient-level data will be stored and analyzed on a secure LAC DHS server. Only approved study team members will have access to identifiable data. After study completion, de-identified data will be transmitted to and stored for use by outside researchers at the Data Center of the Research Coordinating Center (RCC), a hub that manages the operational and administrative aspects of the DECIPHeR Alliance [74]. Rather than being transferred to the RCC, patient data will be maintained at LAC DHS and analyzed using code developed by the RCC.

#### Statistical analysis

We will generate baseline descriptive statistics and frequencies for demographic, clinical characteristics, and outcome measures for all participants (providers and patients) by implementation arm and by race or ethnicity. We will use an intent-to-treat approach, i.e., we will include all participating providers from participating clinics. We do not plan interim analyses.

#### Primary outcome analysis

The primary implementation outcome, provider EBP adoption clustered at the clinic level, uses a scoring system to evaluate overall adoption of the modified EBP components (Table 5). Scores range from 0 to 9, with higher scores indicating higher EBP adoption. The primary objective is to compare changes in event rate of provider EBP adoption between usual implementation strategy (Arm 1) and provider- and patient-focused strategies (Arms 2 and 3) at the end of Year 1, using the constrained generalized Poisson mixed-effects model. This analytical approach [50] has been shown to be more efficient than traditional longitudinal models for estimating group differences at follow-up when baseline values are missing. The main fixed effects include a time indicator, a constraint of a common baseline mean across implementation groups, and a group-by-time interaction term. The model also includes random intercepts at bothprovider and clinic levels, as well as a clinic-level time-varying random effect to account for various levels of clustering and repeated measures. Analyses on provider-level implementation outcomes will be based on available data, as we expect low levels of missing data (< 3%) for the primary implementation outcome.

**Table 5:** Provider Evidence-based Practice Adoption Score (Primary Implementation Outcomes)

Component	Data Source	Component eligibility	Scoring
1. Home BP monitor documented or acquired	EHR and clinic surveillance	Patients with uncontrolled HTN empaneled to provider	0: < 30% pts 1: ≥ 30% of pts
2. Home BP readings uploaded onto the patient portal	EHR	Patients with uncontrolled HTN empaneled to provider	0: < 30% of pts 1: ≥ 30% of pts
3. Primary care visit attended within 1–12 weeks of uncontrolled BP^1^	EHR	Patients with uncontrolled HTN empaneled to provider	0: < 50% of pts 1: ≥ 50% of pts
4. HTN clinic nurse visit attended within 1–12 weeks uncontrolled reading^1^	EHR	Patients with uncontrolled HTN empaneled to provider	0: < 30% of pts 1: ≥ 30% of pts
5. Referral of patients to HTN resources (via texts, portal, mailings, phone calls)	EHR, Patient referrals	Patients with uncontrolled HTN empaneled to provider	0: < 50% of pts 1: ≥ 50% of pts
6. Social needs screening conducted	EHR	Patients with HTN empaneled to provider	0: < 50% of pts 1: ≥ 50% of pts
7. CHW assigned and met with patient	EHR	Patient with HTN with identified social needs empaneled to provider	0: < 30% of pts 1: ≥ 30% of pts
8. Uptake of HTN education and training	Course participation and CME requests	All providers	0: 0 class attended 1: ≥ 1 classes attended
9. Combination medications prescribed	EHR	Patients with uncontrolled HTN empaneled to provider	0: < 10% of pts 1: ≥ 10% of pts

*EBP* Evidence-based practice, *BP* Blood pressure, *HTN* Hypertension, *CHW* Community health worker, *EHR* Electronic health records

^1^Time to visit interval will be based on LAC DHS Expected Practice protocols

#### Secondary outcome analyses

To analyze secondary implementation outcomes, we will use a constrained generalized linear mixed-effects model with an appropriate link function to compare the three arms (sequences) at Year 2, using an omnibus test followed by specific comparisons of interest. We will use the same modeling approach to evaluate effects on the health-related outcomes, such as change in hypertension control, at Year 1, and to compare the three arms (sequences) at Year 2. We will also perform adjusted analyses of the above models that include patient- provider-, and clinic-level characteristics.

#### Missing data

We will address missing data for health-related outcomes using multiple imputation (MI) techniques [75, 76], such as MI with fully conditional specification and joint multivariate normal imputation [77]. These techniques have the potential to improve the precision of estimation [78, 79]. We will also examine frequencies and patterns of missing data across the three implementation arms. In sensitivity analyses, we will analyze health-related outcomes with and without the imputed data.

#### Sample size and power considerations

The power analysis was performed based on the primary implementation outcome (count, provider EBP adoption). LAC DHS clinics average 20 providers each, and 27 total clinics are expected to be available. Our proposed sample size is expected to achieve > 90% power (or > 80% power) at 5% level of significance to detect an absolute net difference of 1.46 (or 1.24) when comparing changes in primary outcome between no implementation strategy and any implementation strategies at Year 1. The assumptions used in these calculations were: (a) cohort design, (b) event rate of 3, (b) intra-cluster correlation up to 0.05, (c) correlation of 0.3 between repeated visits of providers, (d) overdispersion of 25%, (e) 2-sided test with type-I error rate of 0.05, and (f) attrition rate up to 3% at the end of Year 1. We used the National Institutes of Health sample size calculator for parallel group-randomized trials [76].

#### Analysis of community engagement activities

We will report descriptive statistics on meeting attendance and analyze meeting discussions using rapid qualitative analysis of meeting transcripts or notes. Guided by EPIS domains, we will develop and use templates to organize and consolidate qualitative data [80]. We will use matrices and other forms of joint displays [81] to integrate quantitative and qualitative data to contextualize implementation outcome findings, partnership, and intervention sustainment.

#### Monitoring study data and patient safety

We will provide a detailed report to the Data and Safety Monitoring Board (DSMB) of NHLBI annually, outlining study and enrollment progress, unanticipated problems, adverse events, and study document updates. The DSMB convenes at least annually and is composed of experts in pulmonary and cardiovascular diseases, implementation science, psychology, pediatrics, health disparities, and statistics, appointed by the Director of NHLBI.

#### Dissemination strategies

We will disseminate our findings to research, community, and policy audiences, including through peer-reviewed publications, scientific meetings, lay press publications, community forums, CAB meetings, policy briefs, and presentations to LAC DHS leadership, staff, and clinics. Authorship will be based on International Committee of Medical Journal Editors recommendations [82].

## Discussion

We will conduct a three-arm, crossover randomized controlled trial to measure the impact of provider- and patient-focused implementation strategies on adoption of hypertension care EBPs that have been culturally and linguistically tailored for racially and ethnically diverse patients at a large safety-net health system [83–88]. We will also examine secondary implementation outcomes (e.g., EBP sustainment) and clinical effectiveness outcomes (e.g., hypertension control and disparities).

We expect our study to produce significant contributions to the implementation science literature through several unique features. First, our interventions used community engagement methods to adapt the Kaiser Bundle according to perceived barriers and facilitators identified by patients, community members, and health system personnel, which can potentially bridge cultural and social gaps between healthcare system and patients [89–96]. Second, our EBPs include social needs screening and community resource referrals, answering the call from the AHA for implementation science studies in cardiovascular health equity that address social determinants of health [23]. Furthermore, there are limited implementation science studies in cardiovascular health equity that examine Asian communities [23]; our collaboration with LAC DHS presents a unique opportunity to work with Filipino American, Chinese American, and Korean American patients. We also critically examine the Chronic Care Model to understand the impact of patient activation versus provider engagement – along with sequencing of the two – on hypertension EBP uptake and clinical outcomes. Finally, our study is grounded in implementation science, using the SIC to track implementation processes and milestones and the COINS to track implementation costs. Findings from our study will help build evidence for implementation of hypertension EBPs in safety-net health systems and similar health systems serving under-resourced populations nationwide.

## Data Availability

Not applicable.

## Abbreviations

ACC/AHA: American College of Cardiology/American Heart Association
CAB: Community action board
CHW: Community health worker
COINS: Cost of Implementing New Strategies
DECIPHeR: Disparities Elimination through Coordinated Interventions to Prevent and Control Heart and Lung Disease Risk
EBP: Evidence-based practice
EHR: Electronic health record
EPIS: Exploration, Preparation, Implementation, Sustainment
HeartBEAT: Heart blood pressure equity action team
HEDIS: Healthcare Effectiveness Data and Information Set
LAC DHS: Los Angeles County Department of Health Services
NCQA: National Committee for Quality Assurance
NIMHD: National Institute on Minority and Health Disparities
PDSA: Plan-do-study-act
RE-AIM: Reach, Effectiveness, Adoption, Implementation, and Maintenance
SIC: Stages of Implementation Completion
